# The Influence of Crystal Anisotropy in Femtosecond Laser Processing of Single-Crystal Diamond

**DOI:** 10.3390/nano15151160

**Published:** 2025-07-28

**Authors:** Guolong Wang, Ji Wang, Kaijie Cheng, Kun Yang, Bojie Xu, Wenbo Wang, Wenwu Zhang

**Affiliations:** 1 College of Mechanical Engineering, Zhejiang University of Technology, Hangzhou 310014, China; wangguolong@nimte.ac.cn; 2 Research Center for Laser Extreme Manufacturing, Ningbo Institute of Materials Technology & Engineering, Chinese Academy of Sciences, Ningbo 315201, Chinayangkun@nimte.ac.cn (K.Y.); xubojie@nimte.ac.cn (B.X.); zhangwenwu@nimte.ac.cn (W.Z.); 3 Zhejiang Key Laboratory of Laser Extreme Manufacturing of Difficult-to-Machine Materials, Ningbo 315201, China; 4 Faculty of Mechanical Engineering & Mechanics, Ningbo University, Ningbo 315211, China

**Keywords:** femtosecond laser, single-crystal diamond, lattice orientation, ablation threshold, graphitization threshold, phase change, surface morphology

## Abstract

The single-crystal diamond (SCD), owing to its extreme physical and chemical properties, serves as an ideal substrate for quantum sensing and high-frequency devices. However, crystal anisotropy imposes significant challenges on fabricating high-quality micro-nano structures, directly impacting device performance. This work investigates the effects of femtosecond laser processing on the SCD under two distinct crystallographic orientations via single-pulse ablation. The results reveal that ablation craters along the <100> orientation exhibit an elliptical shape with the major axis parallel to the laser polarization, whereas those along the <110> orientation form near-circular craters with the major axis at a 45° angle to the polarization. The single-pulse ablation threshold of the SCD along <110> is 9.56 J/cm^2^, representing a 7.8% decrease compared to 10.32 J/cm^2^ for <100>. The graphitization threshold shows a more pronounced reduction, dropping from 4.79 J/cm^2^ to 3.31 J/cm^2^ (31% decrease), accompanied by enhanced sp^2^ carbon order evidenced by the significantly intensified G-band in the Raman spectra. In addition, a phase transition layer of amorphous carbon at the nanoscale in the surface layer (thickness of ~40 nm) and a narrow lattice spacing of 0.36 nm are observed under TEM, corresponding to the interlayer (002) plane of graphite. These observations are attributed to the orientation-dependent energy deposition efficiency. Based on these findings, an optimized crystallographic orientation selection strategy for femtosecond laser processing is proposed to improve the quality of functional micro-nano structures in the SCD.

## 1. Introduction

As an ultimate wide-bandgap semiconductor, the diamond integrates ultrahigh hardness, extreme thermal conductivity, and broad-spectrum transmittance (from ultraviolet to far-infrared) [1], exhibiting irreplaceable strategic values in quantum sensing (NV color centers) [2,3], the thermal management of high-power devices [4,5], and optical windows for extreme environments [6,7]. However, its strong covalent bonding and intrinsic brittleness define it as a typical difficult-to-machine material, posing significant challenges to precision machining. While mechanical machining offers a high material removal rate, it is highly susceptible to cleavage fracture and edge chipping, accompanied by poor surface integrity [8,9]. Chemical etching, despite its suitability for fine adjustments or auxiliary procedures, suffers from low efficiency and potential environmental risks [10,11]. To address these processing bottlenecks, laser technology has been incorporated into diamond machining [12,13,14,15].

Although the nanosecond laser can achieve a high material removal rate, its long pulse duration leads to remarkable thermal accumulation, causing extended heat-affected zones, micro-crack initiation, and uncontrolled graphitization, which severely deteriorate the optical and mechanical properties [16,17]. In contrast, the femtosecond laser enables “cold machining” via ultra-short pulse width and ultra-high peak power density. The pulse duration is much shorter than the electron–phonon relaxation time, leading to highly localized energy deposition that effectively suppresses thermal diffusion, reduces the recast layer thickness and micro-crack formation, and achieves near-damage-free processing [18,19,20]. Femtosecond lasers have higher processing accuracy and a lower heat-affected zone compared to traditional tools and nanosecond processing. With a pulse duration falling between the two, picosecond lasers yield a surface quality superior to nanosecond lasers yet inferior to femtosecond lasers. In recent years, significant progress has been made in the study of the impact of the physical parameters (power amplitude, field distribution, wavelength, frequency, etc.) of lasers on processing at the microscale [21,22]. L.K. Nolasco et al. [23] systematically investigated the damage thresholds and incubation curves of the CVD diamond under femtosecond lasers with different wavelengths, expanding the processing understanding from near-infrared to ultraviolet [19]. Matteo Mastellone et al. [24] textured diamond surfaces using two time-delayed and cross-polarized femtosecond pulses to form 2D-LIPPS, significantly enhancing visible light absorption. At a more microscopic level, Han et al. [25] revealed the dynamic graphitization process induced by a femtosecond laser at the atomic scale through high-resolution transmission electron microscopy (HRTEM), providing theoretical foundations for ultra-precision micro-nano machining and functional carbon material development. The refined processing of single-crystal diamonds using femtosecond lasers has found promising applications in the field of functional devices. For instance, Marco Girolami et al. [26] etched LIPSS structures into natural diamonds via femtosecond lasers, converting them into “black” diamonds for the fabrication of solar energy converters, which exhibit excellent absorption exceeding 90% across the solar radiation wavelength range. Patrick S. Salter [27] et al. induced graphite lines inside diamonds through femtosecond lasers, making diamonds applicable for ionizing radiation detectors.

In addition, there are also studies from the perspective of material physicochemical properties. Odake S. et al. [28] compared the laser processing effects of the ultraviolet nanosecond laser on superhard nanocrystalline (NPD) and the single-crystal diamond (SCD), and found that the low thermal conductivity of the diamond leads to more efficient laser energy absorption and a lower ablation threshold, resulting in deeper cutting grooves. Takeyama N. et al. [29] investigated the response of the CVD and HPHT single-crystal diamonds to the nanosecond laser, established processing threshold models for upper/lower surfaces, providing critical process guidelines for laser machining of different types of diamond.

Notwithstanding these advances in the laser machining of diamonds, existing research primarily focuses on material physical properties and process parameter optimization, without considering the anisotropic effects of single crystals. Notably, the unique anisotropy of the single-crystal diamond exerts a critical influence on femtosecond laser processing behavior, yet related studies remain notably insufficient. For example, Berhane M A et al. [30] explored the effects of nanosecond and picosecond laser pulse duration on the micro-nano structure etching of <100>, <110>, and <111> crystal planes, establishing the variation laws of pattern type, surface roughness, and etching rate. Han H. et al. [31] discovered significant crystallographic orientation dependence of graphitization during picosecond laser single-track scanning. While these studies provide important clues for revealing the coupling mechanism between anisotropy and laser processing, the underlying micro-mechanisms still require in-depth exploration. Elucidating the coupling mechanism between the femtosecond laser and diamond anisotropy is the core scientific issue for breaking through precision machining bottlenecks. This work systematically investigates the response differences between <100> and <110> orientations under femtosecond laser processing by constructing an orientation-specific ablation threshold model, combined with in situ Raman spectroscopy and cross-scale topography characterization. These findings will provide theoretical support and technical guidance for the orientation-optimized design of diamond quantum devices and optical components.

## 2. Materials and Methods

### 2.1. Experimental Setup

**Figure 1:**
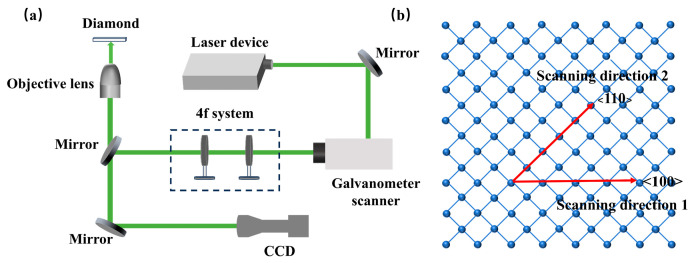
Schematic diagram of processing experiment: (**a**) schematic diagram of the processing equipment; (**b**) relationship between the laser scanning direction and the atomic arrangement of the single-crystal diamond (100) plane.

The femtosecond laser processing system employed in this study is illustrated in Figure 1a. The femtosecond laser (FemtoYL-Green, YSL Photonics, Wuhan, China) features a central wavelength of 515 nm, a pulse duration of 286 fs, and a tunable repetition rate ranging from 25 kHz to 5 MHz. The output beam is linearly polarized with beam quality factors of Mx^2^ = 1.117 and My^2^ = 1.133 (beam roundness: 98.3%), exhibiting a Gaussian energy distribution. The beam is first transmitted through a mirror into a scanning galvanometer, then passes through a 4f optical system, and is finally focused onto the single-crystal diamond surface by a 10× objective lens. For an incident Gaussian beam, the spot radius after focusing by an ideal objective lens can be expressed as Equation (1):

(1) $$w_{0}=\frac{2\lambda f^{\prime }}{\pi w_{i}}$$

Here, $w_{0}$ denotes the radius of the focal spot, λ is the laser wavelength, $f^{\prime }$ represents the focal length of the objective lens of 18 mm, and $w_{i}$ corresponds to the radius of the laser beam incident on the objective lens. By substituting the data, the focused spot diameter is calculated to be 2.32–2.36 μm via Equation (1). Furthermore, the spot diameter was also determined through processing experiments. A precision 3D motion stage was utilized to adjust the *Z*-axis height, and laser scanning was performed on the aluminum alloy to fabricate groove lines. The line width at the narrowest point was measured, yielding a dimension of approximately 2.4 μm under a scanning electron microscope. The machining process is real-time monitored by an imaging system, and after processing, the workpiece morphology and dimensions are preliminarily inspected via a CCD camera, which enhances experimental efficiency.

The instruments used for characterization in this study include the following: scanning electron microscope (SEM, S4800, Hitachi, Tokyo, Japan), confocal micro-Raman spectrometer (Renishaw inVia Reflex, Renishaw, Gloucestershire, UK), transmission electron microscope (Tecnai F20, FEI, Hillsboro, OR, USA), and UV-Vis-NIR spectrophotometer (Lambda 950, Perkin-Elmer, Waltham, MA, USA). The laser wavelength employed in the Raman spectroscopy measurements was λ = 532 nm.

### 2.2. Experimental Method

The equivalent number of laser pulses deposited per unit area, denoted as N, is calculated using Equation (2) [32]

(2) $$N=\frac{2w_{0}f}{v}$$

Here, $w_{0}$ is the waist radius of the focused Gaussian beam (1/e^2^ intensity), f is the laser repetition frequency, and v is the scanning speed. It follows that increasing the scanning speed at a fixed repetition frequency f reduces the number of pulses irradiating each spot. In this experiment, the laser repetition frequency was set to 25 kHz, and the scanning speed was determined to be 100 mm/s to achieve the strict single-pulse condition (N = 1). Figure 1b illustrates the correspondence between the atomic arrangement of the diamond (100) plane and the laser scanning direction. To reveal the effect of crystallographic orientation dependency on the single-pulse ablation threshold, laser scanning was strictly performed along the crystallographic directions shown in the figure, ensuring that the electric field vector was aligned with specific crystal axes.

### 2.3. Experimental Materials

The single-crystal diamond used in this study was synthesized by Zhengzhou Abrasives Grinding Research Institute Co., Ltd. via the chemical vapor deposition (CVD) technique, with dimensions of 9 mm × 9 mm × 0.2 mm. The sample surface exhibits a {100} crystallographic orientation and was double-side polished to a final roughness of approximately 1 nm. Before and after laser irradiation, all samples were ultrasonically cleaned in ethanol for 10 min to remove surface impurities, and all experiments were conducted in a standard atmospheric environment with constant temperature and humidity.

## 3. Results

### 3.1. Surface Morphology

**Figure 2:**
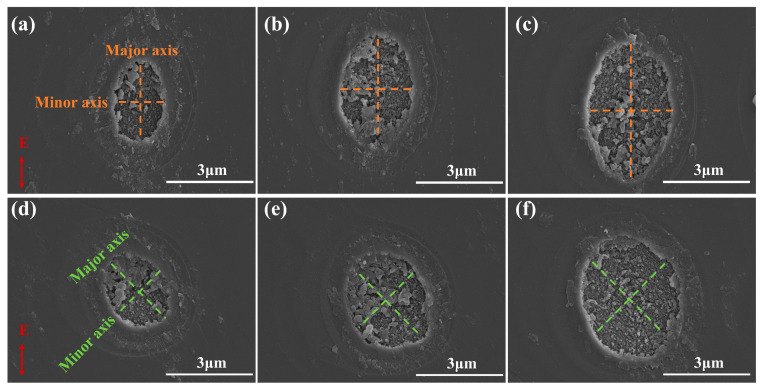
SEM images of ablation pits under different single-pulse energies: (**a**–**c**) pit morphologies of <100> orientation under single-pulse energies of 4.4 μJ, 7.5 μJ, and 11.4 μJ, respectively; (**d**–**f**) pit morphologies of <110> orientation under single-pulse energies of 4.4 μJ, 7.5 μJ, and 11.4 μJ, respectively.

Figure 2 illustrates the evolution of ablation pit morphologies for <100> and <110> orientations under different single-pulse energies, where the red arrow designates the laser polarization direction (i.e., electric field vector direction). In both orientations, the morphology of the ablated hole presents an elliptical shape, with the major and minor axis directions marked in Figure 2. In both orientations, the long axis of the elliptical ablation pit exhibits a significant increasing trend with the increase in pulse energy. However, at the same single-pulse energy, the major axis of pits in <100> orientation is slightly larger than that in <110> orientation. When the single-pulse energy is 4.4 μJ, the major axes of the ablation pits in the <100> and <110> orientations are approximately 2.47 μm and 2.32 μm, respectively. When the single-pulse energy is 7.5 μJ, the major axes of the ablation pits in the <100> and <110> orientations are approximately 3.25 μm and 2.92 μm, respectively. When the single-pulse energy reaches 11.4 μJ, the major axes of the ablation pits in the <100> and <110> orientations are approximately 4.12 μm and 3.52 μm, respectively. As the single-pulse energy increases from 4.4 μJ to 11.4 μJ, the major axis of the <100> crystal orientation elongates by 1.65 μm, whereas that of the <110> crystal orientation increases by 1.2 μm. It is noteworthy that the growth rate of the major axis for the <100> orientation exceeds that for the <110> orientation. From Figure 2, it can be observed that <100>-oriented pits show a typical elliptical shape, and their major axis direction is consistent with the direction of the femtosecond laser electric field vibration. Meanwhile, the <110>-oriented pits approach a circular shape, with their major axis forming an angle of approximately 45° with the laser polarization direction. We analyzed the roundness of pulse ablation pits through eccentricity analysis. The eccentricity is expressed by Equation (3)

(3) $$\mathrm{e}=\frac{c}{a},$$

**Figure 3:**
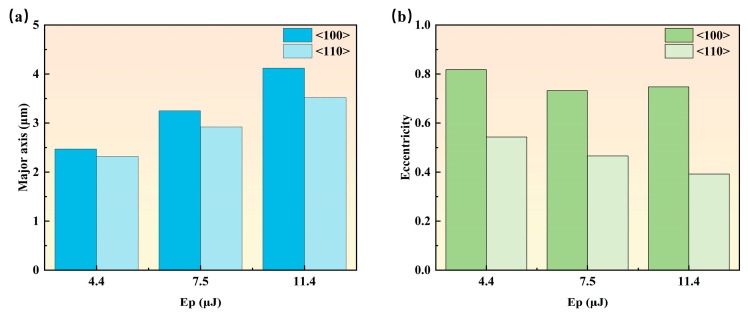
Eccentricity analysis of different crystal orientation ablation pits in Figure 2: (**a**) the length of the major axis; (**b**) eccentricity calculation results.

Here, a is the semi-major axis and c is the semi-focal length. An eccentricity close to 0 indicates a shape closer to a circle. Figure 3 shows that the eccentricity of <100>-oriented pits fluctuate within the range of 0.7–0.8, whereas those of <110>-oriented pits stabilize at 0.4–0.5. Since an eccentricity closer to 0 indicates a shape closer to a circle, this theory explains the rounder morphology of <110>-oriented pits compared to <100> orientation well. Additionally, it is found that with the increase in single-pulse energy, the eccentricity of pits in the <110> lattice orientation shows a consistent decreasing trend, indicating that the pit shape gradually transforms into a circle.

**Figure 4:**
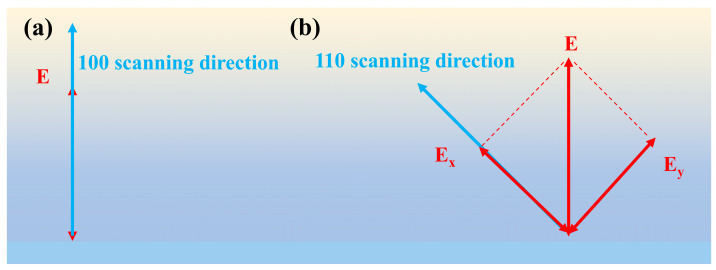
Energy action mechanism of a vector electric field: (**a**) electric field and scanning direction of <100> orientation; (**b**) electric field and scanning direction of <110> orientation.

During the experimental setup, the angle between the laser scanning direction and the material lattice orientation is kept constant. By changing the angle between the femtosecond laser electric field vibration direction and the crystal orientation, the influence of different crystal orientations on laser ablation is analyzed. When the orientation of the target carbon atoms is <100>, the laser scanning direction is adjusted along the <100> crystal orientation. Correspondingly, the polarization direction of the laser electric field is parallel to the <100> crystal orientation (as shown in Figure 4a), which helps to deposit energy along the scanning direction, thereby forming elliptical pits elongated along the polarization axis. In contrast, when the orientation of the target carbon atoms is <110>, the laser scanning direction is adjusted along this direction, and the polarization direction of the laser electric field forms a 45° angle with <110> crystal orientation (Figure 4b). The energy deposition mechanism is analyzed through vector decomposition in Figure 4. The electric field vector E is decomposed into a component parallel to the <110> crystal orientation ($E_{x}$) and its perpendicular component ($E_{y}$), with the formula $E_{x}=E_{y}=E\cdot \sin45^{{}^{\circ}}$ showing that the amplitudes of both decomposed components are smaller than the original electric field vector |E|. Under the same pulse energy, the effective applied electric field along the <110> direction is reduced, thus weakening the energy deposition effect of the electric field along the <110> crystal orientation. Therefore, for laser machining in the <110> orientation, the major axis lengths of the formed ablation pits are smaller than those in the <100> orientation, which reasonably explains why the shape of ablation pits under the <110> orientation approximates a circular shape (Figure 2d–f).

### 3.2. Ablation Threshold

**Figure 5:**
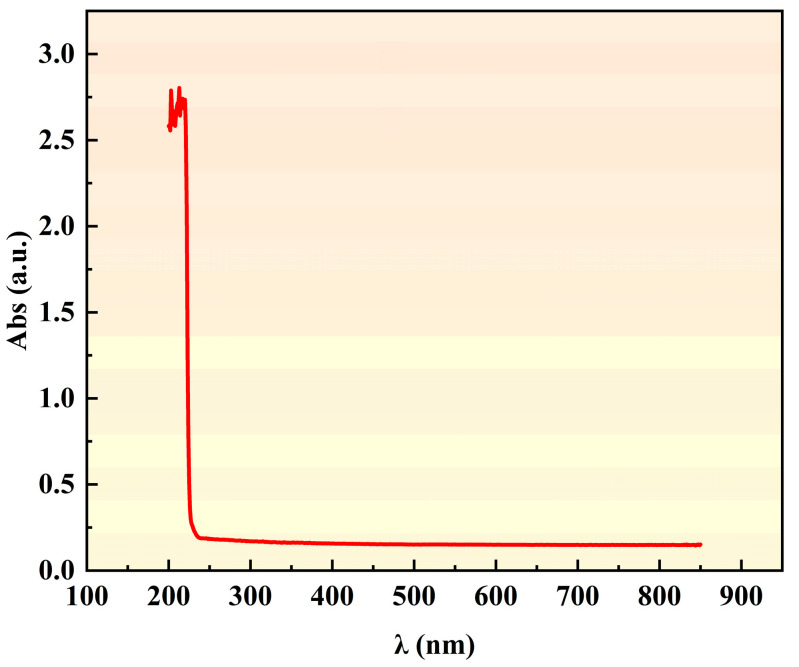
UV–VIS absorption spectra of the diamond used in this study.

As a typical wide-bandgap transparent hard-brittle material (bandgap Eg ≈ 5.5 eV), the study of the ablation threshold for diamonds must first clarify its intrinsic absorption characteristics toward laser wavelength. To this end, ultraviolet-visible absorption spectroscopy characterization was performed on the single-crystal diamond samples (Figure 5). The test results show that in the vacuum ultraviolet band (λ < 255 nm), the material exhibits significant light absorption (absorption coefficient α ≈ 2.5–2.8 cm^−1^), while the absorption spectrum in the visible band (400–700 nm) tends to be flat, with the absorption coefficient stabilized at approximately 0.1 cm^−1^. The absorption rate of the diamond at the wavelength used in this experiment (515 nm) is extremely low (α 532 nm ≈ 0.1 cm^−1^), and the photon energy (hν ≈ 2.33 eV) is much lower than its bandgap, resulting in almost no linear light absorption. Therefore, it is necessary to induce nonlinear absorption processes (such as multiphoton absorption) through high single-pulse energy to achieve material ablation, which is consistent with the typical ablation behavior of wide-bandgap transparent materials [33,34].

The inherent crystallographic anisotropy of diamonds leads to subtle differences in the single-pulse ablation threshold among different orientations. To systematically quantify this effect, the ablation pit diameter method was used to measure the ablation thresholds of different orientations—the D-squared method was employed to analyze the pit sizes under various pulse energies (part of the morphological evolution is shown in Figure 2), followed by calculations based on Equation (4) [25]:

(4) $$D^{2}=2w_{0}^{2}\left[\ln\left(E_{p}\right)-\ln\left(E_{th}\right)\right]$$

**Figure 6:**
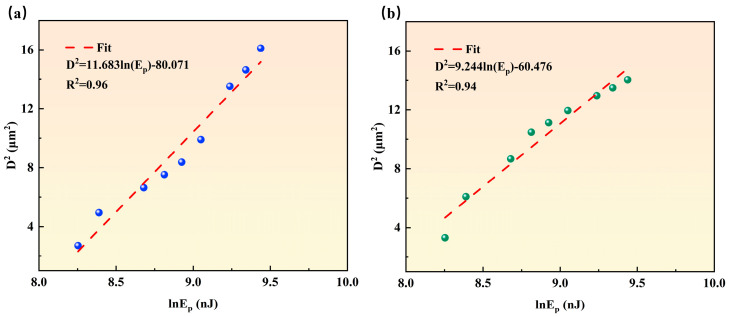
Functional relationship between the square of the ablation pit diameter and natural logarithm of single-pulse energy: (**a**) <100> orientation; (**b**) <110> orientation.

Here, D is the ablation pit diameter, $w_{0}$ is the waist radius of the focused Gaussian beam (1/e^2^ intensity), $E_{p}$ is the single-pulse energy, and $E_{th}$ is the damage threshold energy. The waist radius $w_{0}$ was determined by the linear fitting of $D^{2}$ versus $\ln\left(E_{p}\right)$ (as shown in Figure 6), with $w_{0}$ extracted from the fitting slope and the damage threshold energy $E_{th}$ from the intercept. The single-pulse ablation threshold $F_{AT}$ was then derived using Equation (5)

(5) $$F_{AT}=\frac{2E_{th}}{\pi w_{0}^{2}}$$

Figure 6 depicts the functional relationship between the square of the ablation pit diameter and the natural logarithm of single-pulse energy for different orientations, with the functional expressions and goodness-of-fit R^2^ values marked in the figure. For the <100> orientation in Figure 6a, the waist radius $w_{0}$ is calculated as 2.41 μm from the fitting slope, and the damage threshold energy $E_{th}$ is obtained as 947.31 nJ from the intercept, yielding a single-pulse ablation threshold of 10.32 J/cm^2^ via Equation (3). Similarly, for the <110> orientation in Figure 6b, $w_{0}$ = 2.14 μm and $E_{th}$ = 693.81 nJ are derived, corresponding to an ablation threshold of 9.55 J/cm^2^. The presence of the multi-pulse effect leads to a reduction in the ablation threshold [35]. In practical laser machining applications, such as the etching of two-dimensional patterns including drilling and scribing, multi-pulse operations are predominantly employed. However, machining under multi-pulse conditions typically necessitates the consideration of the thermal accumulation effect, which can be addressed through the optimization of laser parameters such as energy, processing speed, and frequency. The fitting curves exhibit high goodness-of-fit with R^2^ = 0.96 and 0.94, respectively, validating the result accuracy. Comparison of ablation thresholds reveals that the <110> orientation has a slightly lower threshold than <100>, which is attributed to the perfect tetrahedral symmetry of sp^3^ bonds on (100) planes that necessitates higher energy for rupture. In contrast, the more pronounced bond angle distortion on (110) planes lead to bond softening, thereby reducing the ablation energy barrier [33].

### 3.3. Graphitization Threshold

**Figure 7:**
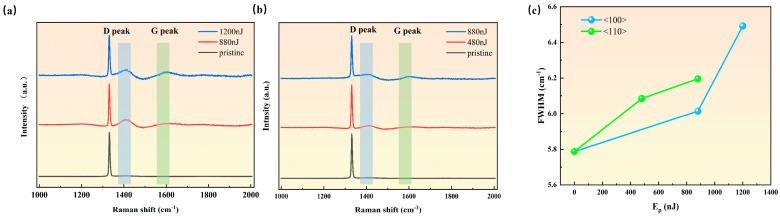
Raman spectra under different single-pulse energies: (**a**) <100> orientation; (**b**) <110> orientation; (**c**) the FWHM of different crystal orientations.

Graphitization, a critical phase transition in the laser machining of diamonds, exhibits pronounced crystallographic orientation dependency that is fundamental to precision processing. Raman spectroscopy from single-pulse energy gradient experiments (Figure 7) reveals that the <100> orientation initially exhibits a distinct D-band (~1350 cm^−1^) occurring at 880 nJ—characteristic of defects, amorphous structures, or low-symmetry carbon configurations, while the G-band (~1582 cm^−1^, corresponding to sp^2^-hybridized graphite vibrations) remains weakly intense. Along the <100> crystal orientation, compared to the unprocessed diamond sample, the processed diamond peak exhibits a red shift, with the magnitude of the red shift being identical at 880 nJ and 1200 nJ (from 1330.96 cm^−1^ to 1330.86 cm^−1^). In contrast, the G peak shows a certain degree of blue shift relative to its standard position (1582 cm^−1^), shifting to 1588.16 cm^−1^ at 1200 nJ. A comparison between Figure 7a and Figure 7b reveals that the direction and magnitude of the shifts in the processed diamond peak and G peak are insensitive to crystal orientation.

When the ablation craters undergo rapid cooling after laser irradiation, their contraction is constrained by the surrounding unprocessed regions. The lattice in the laser-processed area is pulled by the more stable surrounding lattice, generating tensile stress, which results in a slight increase in the C-C bond length within the diamond structure. This change reduces the frequency of the lattice vibration mode, which is manifested in the Raman results as a shift in the processed diamond peak to lower wavenumbers (red shift). The blue shift in the G peak, on the other hand, is attributed to the compressive stress experienced by the newly formed graphite phase. During femtosecond laser irradiation, localized high temperatures induce partial graphitization of the diamond in the irradiated area (conversion from sp^3^ to sp^2^ hybridization). As a newly generated phase, the graphite phase differs from the surrounding diamond in terms of morphology and coefficient of thermal expansion. As the crater region cools, the surrounding diamond matrix cools and contracts first, exerting pressure on the newly formed graphite domains. This compressive stress compresses the C-C bonds involved in stretching vibrations within the graphite lattice planes. The reduced bond length leads to an increased vibration frequency, which is reflected in the Raman results as a shift in the processed G peak to higher wavenumbers (blue shift).

**Figure 8:**
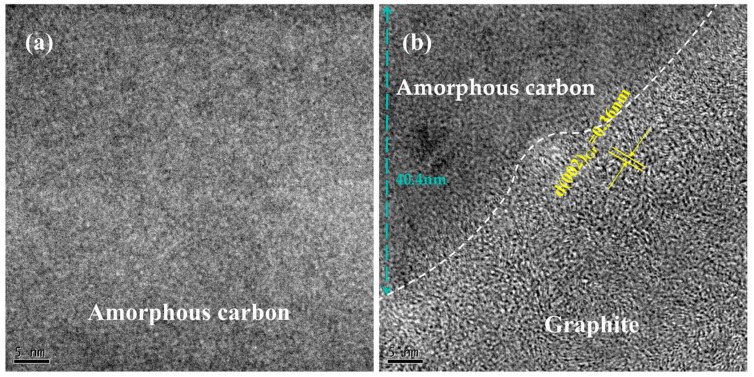
TEM test results: (**a**) TEM images of amorphous carbon; (**b**) TEM images of graphite.

The FWHM of the diamond Raman peak is presented in Figure 7c. The full width at the half maximum (FWHM) of the diamond Raman peak is a crucial figure of merit for crystal quality. The FWHM of the unprocessed sample was 5.78 cm^−1^. Along the <100> crystal orientation, as the laser energy increased from 880 nJ to 1200 nJ, the FWHM rose from 6.01 cm^−1^ to 6.49 cm^−1^. Meanwhile, along the <110> crystal orientation, with the laser energy increasing from 480 nJ to 880 nJ, the FWHM increased from 6.08 to 6.19 cm^−1^. These results indicate that with the increasing laser pulse energy, the diamond crystal contains a higher density of defects, accompanied by a reduction in lattice order (Figure 8). Consequently, the phonons experience more frequent scattering, leading to a shortened phonon lifetime, which in turn results in the broadening effect of the diamond Raman peak.

To elucidate the atomic origins of these Raman features, transmission electron microscopy (TEM) characterization was performed on diamond samples (Figure 8). Specifically, Figure 8a depicts amorphous carbon structures, directly correlating with the disordered/low-symmetry phases represented by the D-band, whereas Figure 8b illustrates layered graphitic phases with interplanar spacing of ~0.36 nm, consistent with the sp^2^-hybridized vibration mode of the G-band. Notably, when the pulse energy increases to 1200 nJ, the G-band intensity escalates significantly, indicating the formation of ordered sp^2^ phases. In contrast, the initial display of the D band in the <110> direction occurred at 480 nJ, with a pronounced G-band enhancement at 880 nJ. The energy density at which the D-band significantly appears and the G-band initially emerges is defined as the graphitization energy density threshold $F_{GT}$. The graphitization threshold energies under different orientations are substituted into Equation (6)

(6) $$F_{GT}=\frac{E_{GT}}{\pi w_{0}^{2}}$$

Here, $E_{GT}$ is the threshold energy for the initiation of graphitization measured experimentally.

The graphitization threshold energies under different orientations are substituted into Equation (6); the results show that the graphitization energy density thresholds are 4.79 J/cm^2^ for <100> orientation and 3.31 J/cm^2^ for <110> orientation, exhibiting the same crystallographic dependency as the ablation thresholds (i.e., <110> < <100>). Notably, whereas the <110> ablation threshold is only 8% lower than <100>, the graphitization threshold shows a 30% reduction, highlighting a more pronounced orientation sensitivity. The small size of the ablation pit (Figure 2) indicates that the energy density of the laser acting on the SCD surface is high, making it easier to achieve the ablation threshold and graphitization threshold in the <110> orientation. This means that the crystal structure of this orientation is more sensitive to laser thermal effects and electron excitation (Figure 4), making it easier for local carbon atoms to undergo ablation and recombination, leading to a higher likelihood of graphitization.

Figure 8 reveals the nanoscale resolution structure of the irradiated diamond and displays different carbon structures during the single-pulse induced phase transition process. There is no typical long-range ordered diffraction spot crystal structure on the surface (Figure 8a), indicating that the ablation pit phase transition in the extremely shallow range of the surface (~40 nm) is composed of disordered amorphous carbon. These amorphous carbons also confirm the appearance of the D peak in Raman spectroscopy (Figure 7). Amorphous carbon is accompanied by the appearance of ordered layered crystal structures. Figure 8b shows a narrow lattice spacing of 0.36 nm, corresponding to the interlayer (002) plane of graphite. These graphite sheets are arranged in different directions, with some showing slight curvature. The appearance of the graphite phase further confirms the increase in the G-peak in Raman spectroscopy.

**Figure 9:**
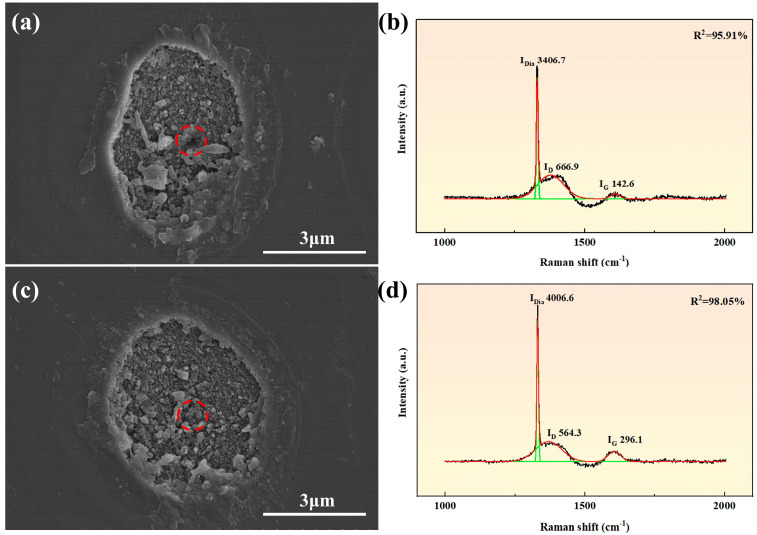
SEM images and Raman fitting spectra at 12.56 μJ: (**a**,**b**) <100> orientation; (**c**,**d**) <110> orientation.

The change in the phase transition degree is further calculated. Scanning electron microscopy (SEM) was used to observe samples of different orientations processed at 12.56 μJ (Figure 9). Raman spectra of the regions marked by red dashed circles in Figure 9a,c show the coexistence of the diamond peak (1332 cm^−1^), D-band, and G-band, confirming that the modified layer is a mixed structure of sp^3^ diamond phase, sp^2^ graphite phase, and disordered carbon. The graphitization degree is characterized by the order parameter I_G_/I_D_ (the ratio of G-peak to D-peak), where a larger ratio indicates a higher graphitization order. The results were obtained by Raman spectrum fitting, with original data in black, and goodness-of-fit values R^2^ (95.91%, 98.95%) labeled. The green curves correspond to the fitted profiles of individual peaks, while the red one represents the fitted curve of the overall dataset. The fitting process was conducted in Origin software (Origin 2024) using the Gaussian method. As shown in Figure 9, the I_G_/I_D_ ratio is ~0.21 for <100> orientation and ~0.52 for <110> orientation, indicating that <110> orientation exhibits stronger graphitization tendency and order [36]. This is related to the amplitude of the graphitization threshold. A lower graphitization threshold means that less energy is required under this crystal orientation during laser interaction, making the material more prone to graphitization. Specifically, when the laser energy reaches or exceeds this threshold, the originally disordered and chaotic arrangement of carbon atoms caused by laser ablation undergoes reorganization in localized areas, forming an ordered layered graphite structure (Figure 8b). This indicates that the material is more sensitive to laser energy in this direction, thereby promoting the initiation and development of the graphitization process. Subtle differences exist in the graphitization degree among different crystal orientations, and this intriguing phenomenon can provide a theoretical basis for enhancing the sensitivity of detectors based on the graphitization transformation of a single-crystal diamond [27]. In addition, to improve optical performance, high-temperature annealing treatment in an inert atmosphere (such as Ar or N_2_) can promote selective diffusion and volatilization of the graphite phase by utilizing the vapor pressure difference between diamond and graphite. Meanwhile, amorphous carbon may be transformed into more stable structures through atomic rearrangement.

## 4. Conclusions

Based on the crystallographic anisotropy of the SCD, this study systematically quantified the single-pulse ablation and graphitization energy density thresholds for <100> and <110> orientations using the ablation pit diameter method, yielding the following key conclusions:

(1) The single-pulse ablation pit is elliptical, and its shape is influenced by the angle between the femtosecond laser’s electric field vibration and the lattice orientation. When the angle is 45 degrees from the <110> orientation, the ablation pit resembles a standard circle more closely.
(2) There are differences in ablation thresholds for different orientations, with the threshold for <110> orientation (9.55 J/cm^2^) slightly lower than that for <100> orientation (10.32 J/cm^2^).
(3) Graphitization thresholds follow the same orientation trend as ablation thresholds, with <110> orientation (3.31 J/cm^2^) demonstrating a 30% reduction compared to <100> orientation (4.79 J/cm^2^). Furthermore, the graphitization order parameter IG/ID (0.52) for <110> is significantly higher than that for <100> (0.21), indicating that <110> orientation exhibits a stronger graphitization tendency and order. Moreover, amorphous carbon and a narrow lattice spacing of 0.36 nm are observed under TEM, corresponding to the interlayer (002) plane of graphite.

These results provide valuable guidance for orientation selection in diamond laser processing; diamonds with <110> orientation are more prone to graphitization, which is beneficial for the manufacture of low damage micro-nano graphite structures, while <100> orientation is more suitable for high-precision ablation applications. Future work will integrate theoretical modeling and simulation to deeply explore the interaction mechanism between the femtosecond laser and diamond micro-phases, offering more valuable guidance for experimental research and process optimization.

## Data Availability

The original contributions presented in the study are included in the article; further inquiries can be directed to the corresponding author.
